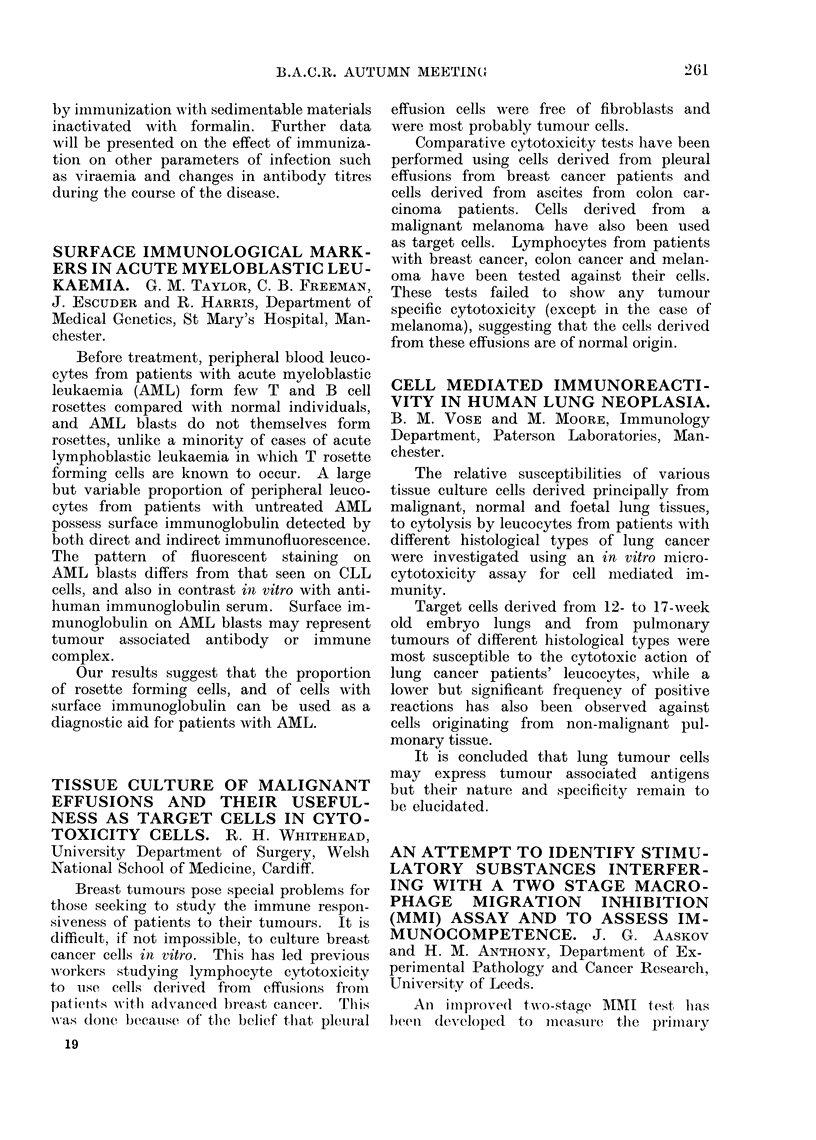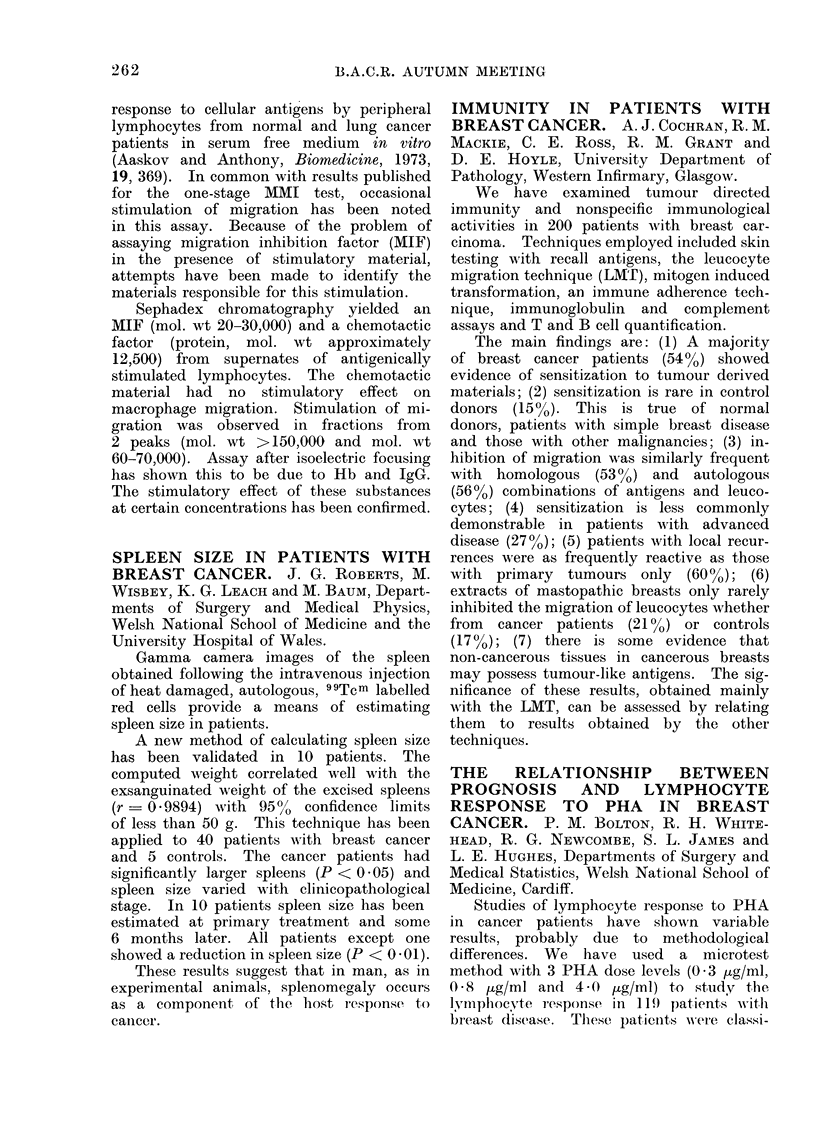# Proceedings: An attempt to identify stimulatory substances interfering with a two stage macrophage migration inhibition (mmi) assay and to assess immunocompetence.

**DOI:** 10.1038/bjc.1975.42

**Published:** 1975-02

**Authors:** J. G. Aaskov, H. M. Anthony


**AN ATTEMPT TO IDENTIFY STIMULATORY SUBSTANCES INTERFERING WITH A TWO STAGE MACROPHAGE MIGRATION INHIBITION (MMI) ASSAY AND TO ASSESS IMMUNOCOMPETENCE.** J. G. AASKOV and H. M. ANTHONY, Department of Experimental Pathology and Cancer Research, University of Leeds.

An improved two-stage MMI test has been developed to measure the primary response to cellular antigens by peripheral lymphocytes from normal and lung cancer patients in serum free medium *in vitro* (Aaskov and Anthony, *Biomedicine*, 1973, **19**, 369). In common with results published for the one-stage MMI test, occasional stimulation of migration has been noted in this assay. Because of the problem of assaying migration inhibition factor (MIF) in the presence of stimulatory material, attempts have been made to identify the materials responsible for this stimulation.

Sephadex chromatography yielded an MIF (mol. wt 20–30,000) and a chemotactic factor (protein, mol. wt approximately 12,500) from supernates of antigenically stimulated lymphocytes. The chemotactic material had no stimulatory effect on macrophage migration. Stimulation of migration was observed in fractions from 2 peaks (mol. wt >150,000 and mol. wt 60–70,000). Assay after isoelectric focusing has shown this to be due to Hb and IgG. The stimulatory effect of these substances at certain concentrations has been confirmed.